# The utility of post-test newborn blood spot screening cards for epigenetic association analyses: association between *HIF3A* methylation and birth weight-for-gestational age

**DOI:** 10.1038/s10038-019-0621-5

**Published:** 2019-05-29

**Authors:** Tay Zar Kyaw, Seiji Yamaguchi, Chihiro Imai, Marina Uematsu, Noriko Sato

**Affiliations:** 1 Department of Epigenetic Epidemiology/Molecular Epidemiology, Medical Research Institute, Tokyo Medical and Dental University, 1-5-45, Yushima, Bunkyo-ku, Tokyo, 113-8510 Japan; 2 Department of Pediatrics, Shimane University School of Medicine, Izumo, Shimane 693-8501 Japan

**Keywords:** Population screening, Epidemiology, Preventive medicine

## Abstract

Identification of disease-associated epigenetic markers in early life might be useful for pre-emptive intervention to prevent diseases. Epigenome-wide association analyses using newborn blood spot screening cards are an anticipated field of research in Japan. Here, in this study, post-test dried blood spot (DBS) samples were anonymized, with only three attributes of gender, gestational age, and birth weight identified. We isolated DNA from DBS (*n* = 300) archived for more than 3 years. The median DNA yield (ng) per individual was 429 (interquartile range 300–565). In a model epigenetic analysis, we conducted a confirmative study on the known association between birth weight and hypoxia-inducible factor 3A (*HIF3A*) gene methylation. DNA methylation levels and *cis*-acting SNP genotypes (rs8102595 and rs3826795) were measured using EpiTYPER and Taqman assays, respectively. *HIF3A* methylation was positively associated with birth weight-for-gestational age centile (*p* = 0.021). While *HIF3A* methylation was associated with *cis*-genotypes (rs8102595, *p* = 2.08E−13; rs3826795, *p* = 3.63E−09), the association with birth weight centile was retained after adjusting for *cis*-genotypes (*p* = 0.029). Thus, we successfully reproduced the results reported previously by others, and demonstrated the usefulness of archived DBS in secondary use for epigenetic association analyses.

## Introduction

The burden of noncommunicable diseases such as cardiovascular diseases and metabolic disorders is increasing worldwide. Especially in super-aging Japan, preemptive medicine is an ultimate goal to ensure a healthy society. Based on a theoretical framework referred to as the “Developmental Origin of Health and Disease (DOHaD)”, it has been recognized that re-establishment of our social systems is needed to collect health-related phenotype and molecular data from infancy and throughout life [1]. Several lines of evidence have shown that disease-associated epigenetic alteration can be detected before clinical diagnosis [2–6]. Even though currently lacking evidence for their causal relationship, disease-related epigenetic markers are regarded as important clinical biomarkers. In some cases, it was found that DNA methylation at birth is significantly associated with phenotypes in later life, including, for example, child obesity and psychiatric conditions at the age of 5–7 years [7, 8]. The utility of newborn blood spot screening cards has been recognized important worldwide to implement longitudinal epigenome-wide association studies (EWAS) [9, 10]. To our knowledge, there are two EWAS using post-test newborn screening cards. The one used the cards retrieved from California Department of Public Health [11] and the other from Danish Neonatal Screening Biobank [12]. In Japan, neonates are routinely screened for inborn errors of metabolism and other congenital disorders by using a small portion of blood on the card, but the card is usually discarded after completion of such screening tests. Secondary use of the post-test dried blood spot (DBS) would be a beneficial strategy for the investigation of neonatal genomic and epigenomic features in life course health care; however, there has been no detailed investigation of the practical secondary usage of newborn blood spot screening cards in Japan.

The aim of this study was to demonstrate the effectiveness of the secondary usage of post-test DBS in Japan for epigenetic analyses. We assessed the quality and quantity of the remaining blood DNA extracted from the archived cards of anonymized newborns in the general population. As a model target site for DNA methylation analysis, we selected the hypoxia-inducible factor 3A (*HIF3A*) gene, whose methylation levels were previously reported in overseas countries to be associated with birth weight [7, 13]. Pan et al. [13] measured *HIF3A* DNA methylation in the umbilical cord and showed positive association with birth weight. According to this report, 10% increase in cord tissue methylation corresponded to an ~3 g increase in birth weight. The methylation sites were the same as the adult BMI-associated CpGs, which was originally reported by Dick et al. [14]. In both newborn and adult cohorts, two single nucleotide polymorphisms (SNPs), rs8102595 and rs3826795, had independent association with *HIF3A* DNA methylation [13, 14]. However, these two SNPs associated neither with birth weight nor with BMI [13, 14]. In the Australian large randomized controlled DOMInO trial of the EPiSCOPE project, DNA methylation in neonatal blood spots, obtained within the first few days after birth, was measured to investigate its relationship to metabolic outcome in childhood at 5 years of age. As a collateral result of this project, it was reported that birth weight was associated with neonatal methylation levels at 136 regions including a *HIF3A* region [7]. Because these reports indicated the plausible association between birth weight and *HIF3A* methylation, we further confirmed this association using newborn blood spot screening cards in Japan. Our study may provide useful information for future longitudinal studies involving a larger cohort in order to identify disease-related epigenetic marks at birth using newborn blood spot screening cards.

## Methods

### Study population

The anonymized blood spot cards of newborns in the general population with no abnormalities detected by screening tests, and archived for more than 3 years at −20 °C were provided by a neonatal mass screening center at the Shimane University. Only three attributes (gender, gestational age, and birth weight) were identified, ensuring sample donor anonymity. Sample characteristics are shown in Table 1. There were no missing attribute data except for one instance of absence of gestational age. Among 300 samples, 283 (94.6%) cases were full-term deliveries. Because of the retrospective and anonymous nature of the samples, the requirement for informed consent was waived. In a calibration experiment (Table S1), we also collected a blood sample from a healthy volunteer. Written informed consent was obtained from the volunteer. Study approval was obtained from the Ethics Committee of Shimane University School of Medicine (approval no. 1747), and the Institutional Review Board of the Medical Research Institute, Tokyo Medical and Dental University (approval no. 2014-014).

**Table 1:** Sample characteristics (*n* = 300)

	Median (IQR), N (%) or mean (SD)
Gestational age (weeks)^a^	39.0 (38.0–40.0)
Child gender (male), *N* (%)	167 (55.7)
Birth weight (g), mean (SD)	3003 (380)

^a^One data point was missing for gestational age

### Genomic DNA preparation

On the newborn blood spot screening card, in principle, there was a blood spot circle 10 mm in diameter, with one punch (3 mm in diameter) cored out for the previous screening tests. The remaining blood area was above 71 mm^2^. For isolation of DNA, eight punches (57 mm^2^ area) each were taken from cards of 300 children. The punches were transferred into NucleoSpin Forensic Filter tubes (Macherey-Nagel, Duren, Germany) and incubated at 56 °C for 1.5 h with shaking in 620 μl of lysis solution containing proteinase K (components of the GenSolve DNA Recovery Kit; GenTegra, Pleasanton, USA). After centrifugation for 5 min at greater than 16300 *g*, 20 µl of recovery solution B (GenTegra) was added to the filtrated lysate. After mixing with 600 µl of ethanol, the lysate was loaded onto a QIAamp micro column (QIAGEN, Hilden, Germany). Genomic DNA was washed on the column and eluted onto a DNA LoBind tube (Eppendorf, Hamburg, Germany) with 100 µl of UltraPure DNase/RNase-free distilled water (ThermoFisher Scientific, Waltham, USA). For the fresh blood sample from a healthy volunteer, buffy coat was prepared and genomic DNA was subsequently extracted using a QIAamp DNA mini kit (QIAGEN). According to Beyan et al., there are little methylomic differences (*R*^2^ = 0.99, Pearson’s) between fresh blood and DBS [9, 15]. Therefore, we used fresh blood instead of DBS for a calibration experiment. Genomic DNA double-stranded DNA (dsDNA) concentration was determined using Quant-iT PicoGreen dsDNA Reagent (ThermoFisher Scientific).

### Genotyping

Genotyping of two *HIF3A* polymorphisms (rs8102595 and rs3826795) was performed by allelic discrimination analysis (the Lightcycler 480 System; Roche, Basel, Switzerland), using TaqMan SNP genotyping assay probes (ThermoFisher Scientific). Genotyping rate was 100% (*n* = 300) and genotype frequencies were found to be in Hardy–Weinberg Equilibrium (HWE) (*P*-value of the HWE test >0.05). The minor allele frequencies of rs8102595 and rs3826795 in neonates were 0.22 (G) and 0.38 (G), respectively. The observed r-squared and D′ measures of linkage disequilibrium between rs8102595 and rs3826795 were 0.28 and 0.78, respectively. These values were similar to those reported in 1000 Genomes JPT, and/or iJGVD databases (https://ijgvd.megabank.tohoku.ac.jp/).

### The quantitative DNA methylation assay using mass spectrometry-based EpiTYPER

Quantitative DNA methylation assays using the EpiTYPER platform and data processing were conducted as described by Suchiman et al. [16]. In brief, genomic DNA (typically 250 ng) was subjected to sodium bisulfite modification using an EZ DNA Methylation Kit (Zymo Research, Irvine, CA, USA), according to the manufacturer’s protocol (Agena Bioscience, San Diego, CA, USA). The sequence corresponding to the *HIF3A* region (GRCh37/h19, chr19:46,801,504-46,801,793) was amplified by PCR using the primers 5′-aggaagagagTTTGGTTTTGGGTTTAATAAGGAAT-3′ and 5′-cagtaatacgactcactatagggagaaggctAAAATATTAAAAACCCACTCACCATC-3′ in 10 μl reactions (95 °C for 15 min; 45 cycles of 95 °C for 20 s, 56 °C for 30 s, and 72 °C for 1 min; and final extension of 72 °C for 3 min). PCR was performed using 10 ng of bisulfite-converted DNA per reaction, in multiple replicates (more than three replicates per subject), and PCR products were treated with shrimp alkaline phosphatase (Agena Bioscience) at 37 °C for 20 min. After confirming successful PCR amplification by agarose gel electrophoresis, a single-stranded RNA product was produced by reverse transcription, and cleaved into fragments with RNase A (Agena Bioscience) at 37 °C for 180 min. The samples were transferred onto a 384 SpectroCHIP, for mass-based fragment separation using a Matrix Assisted Laser Desorption-Ionization Time of Flight platform (Agena Bioscience). The DNA methylation percentage of a given fragment CpG unit was calculated by taking the ratio of the methylated fragment signal to the total signals of methylated and unmethylated fragments, and multiplying by 100. Data exclusion criteria were as follows: (1) CpG units having duplicate or overlapping fragments, (2) missing rate per sample for each CpG unit >0.33, (3) standard deviation per sample for each CpG unit >10% methylation, and (4) CpG units with success rate lower than 80% across subjects. All data passed these quality control checks and were used for analysis in this study.

DNA methylation of five CpG units located adjacent to the transcription start site of *HIF3A* NM_022462/NM_152796 was measured (Fig. S1). Among five CpG units, the first (CpG1.2), second (CpG5), and third (CpG6.7.8) units contained three CpGs, for which probes are present on the Infinium Human Methylation Bead Chip array (CpG1 = cg27146050, CpG5 = cg22891070, and CpG7 = cg16672562).

### Birth weight-for-gestational age centile

Gender-specific birth weight-for-gestational age (BW/GA) centile was calculated using an international standardized reference chart for children’s gender and gestational age [17]. There is a Japanese national reference for BW/GA, but it is gender- and parity-specific [18]. Because the parity information was not accompanying the newborn blood spot screening cards, we could only estimate BW/GA centile by assuming both nullipara and multipara conditions in cases using the national reference. Nevertheless, we found such estimates also similarly associated with *HIF3A* methylation. Therefore, the BW/GA centiles reported in this study are regarded as the representative parameter.

### Statistical analysis

All calculations and graphical presentations were performed using R software (ver.3.5.0). Linear regression models were used to examine the association of *HIF3A* methylation with BW/GA centile, adjusted for child gender. To examine the influence of *cis*-SNPs on methylation levels, we regressed each CpG unit against *cis*-SNPs using an additive genetic model, adjusted for child gender. Effect sizes are reported as percentage increase in methylation for one-unit increment of a predictor variable (10% increase in BW/GA centile; per copy of the allele of effect).

## Results

### DNA yield from post-test DBS

**Fig. 1:**
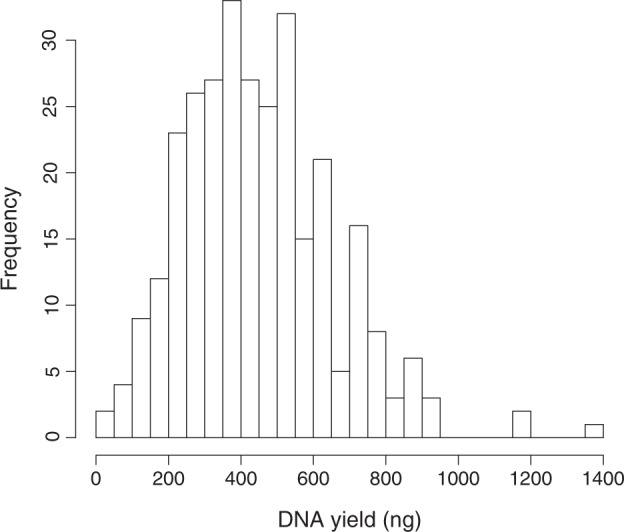
Histogram of DNA yield from archived newborn blood spot screening cards

**Fig. 2:**
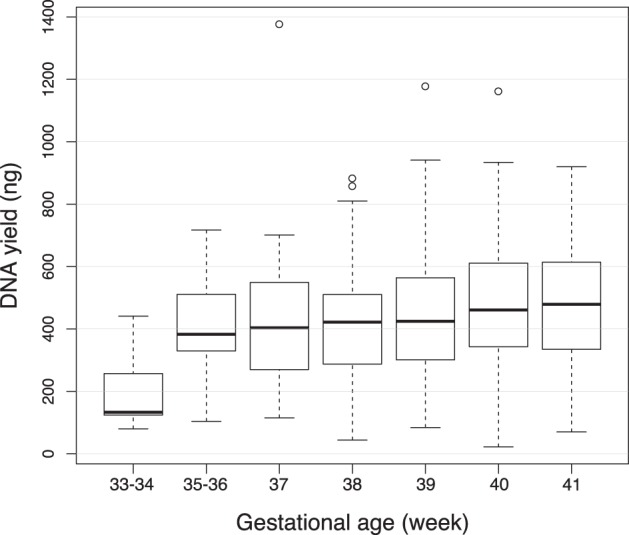
Influence of gestational age on DNA yield

We evaluated the usefulness of heel-prick blood samples for neonatal methylation analysis. A potential issue was that the amount of DNA available for extraction from post-test DBS is limited. However, in our evaluation, the median DNA yield (ng) per individual was 429 (interquartile range 300–565) (Fig. 1). DNA yield increased with gestational age (Fig. 2). In order to validate whether the EpiTYPER assay appropriately measured methylation levels with low DNA yields (less than 200 ng), we tested the assay performance using bisulfite-converted DNA (obtained from an adult volunteer’s blood sample) with starting amounts from 50 to 1000 ng. All EpiTYPER data passed quality control checks (see “Methods” section), even with low quantities of starting DNA. In addition, the measured methylation levels did not vary with the amount of starting DNA (Table S1). Therefore, all DNA samples extracted from neonatal DBSs were included for analysis in this study. Figure S2 shows distribution of methylation levels in each CpG unit. As shown in Table S2, methylation levels of three CpG1.2, CpG5, and CpG6.7.8 units were highly correlated with each other (*R*^2^ = 0.791–0.896), whereas the correlation of these three units with two other units (CpG11 and CpG13.14) were lower (*R*^2^ = 0.425–0.776). We therefore report the mean methylation levels of three CpG units (CpG1.2, CpG5, and CpG6.7.8) in the main text, but show all the data in a supplementary file.

### Association between *HIF3A* methylation and birth weight-for-gestational age

We conducted confirmative association analysis involving neonatal *HIF3A* methylation and birth weight (Tables S3 and 2). Since the reported association was found in full-term delivered children [13], further analyses were performed within full-term pregnancy samples (*n* = 283). Birth weight depends on gestational age, but it was previously shown that the association between *HIF3A* methylation and birth weight is maintained for three binned ranges of gestational age (37.0–38.0, 38.1–39.1, and 39.3–41.4 weeks) [13]. Thus, we calculated the values of BW/GA centiles using standardized growth charts for children’s gender and gestational age [17], and tested for association with *HIF3A* methylation. As shown in Table 2, mean methylation of three *HIF3A* CpG units (CpG1.2, CpG5, and CpG6.7.8) was positively associated with BW/GA centile. Mean methylation of those CpG units increased by 0.21% per 10% increase of BW/GA centile (*p* = 0.021), before adjustment for *cis*-genotypes.

**Table 2:** Association between mean *HIF3A* methylation level and birth weight-for-gestational age (BW/GA) centile in full-term pregnancies (*n* = 283)

	Unadjusted for *cis*-genotypes		Adjusted for *cis*-genotype	
BW/GA centile	Est. (95%CI)	*P* value	Est. (95%CI)	*P* value
	0.21 (0.03–0.39)	0.021	0.18 (0.02–0.34)	0.029

Regression coefficients (Est.) and 95% confidential intervals (CIs) are reported as percentage change in methylation for one unit (10%) increase in birth weight-for-gestational age (BW/GA)

### Association of *HIF3A* methylation and birth weight centile remained significant after adjusting for *cis*-acting SNPs

Two *cis*-SNPs (rs8102595 and rs3826795) were strongly associated with *HIF3A* methylation (Tables 3 and S4). However, neither SNP was associated with BW/GA centile (Table S5). Based on the diplotype distribution, rs8102595-rs3826795 haplotype frequencies were estimated: A-A, 0.59; A-G, 0.19; G-A, 0.03; G-G, 0.19. Those values departed from the expected frequency under linkage equilibrium assumption, indicating linkage disequilibrium (D′ = 0.78) between these two SNPs. The other measure of linkage disequilibrium, r-squared was low (0.28). Effect alleles of rs8102595 and rs3826795, respectively, had positive effects on *HIF3A* methylation as shown in Fig. S3. Even after adjusting these two *cis*-SNPs, association of *HIF3A* methylation and BW/GA centile remained significant. Mean *HIF3A* methylation increased by 0.18% per 10% increase of BW/GA centile (*p* = 0.029, Table 2).

**Table 3:** Association between mean *HIF3A* methylation and *cis*-genotypes (*n* = 283)

	Est. (95%CI)	*P* value
rs8102595^a^	2.83 (2.11–3.55)	2.08E−13
rs3826795^b^	1.98 (1.34–2.62)	3.63E−09

Regression coefficients (Est.) and 95% confidential intervals (CIs) are reported as percentage change in methylation per copy of the effect alleles G^a^ and G^b^

## Discussion

We evaluated the usefulness of post-test DBS for genotyping and DNA methylation analysis. As previously described [10], epigenetic analysis using newborn blood spot screening cards still needs to overcome issues of optimization of the amount and quality of DNA extracted from archived cards. Beyanet al. [15] conducted a proof-of-principle epigenome-wide pilot study using umbilical cord blood spots on Guthrie cards. The yield of DNA from 6 mm diameter spots (28 mm^2^ area) in their experiments was 200 ng, on average. In contrast, our yields were above 400 ng from blood spots of 56 mm^2^ area. The number of leukocytes is usually high in cord blood, whereas peripheral blood leukocytes numbers decrease with increasing postnatal days [19]. As a result, the DNA yield in our study was greater than expected. The post-test DBS generally retained sufficient amounts of DNA to conduct both genome-wide and epigenome-wide studies, which usually require 200 ng per assay. It is noteworthy that the DNA yield tended to be reduced when the gestational age was below 34 weeks, suggesting that the amount of DNA extracted from the cards of preterm births may not always reach the required amount for the complete analyses desired. We showed the reliability of quantitative methylation assays using small amounts of template DNA for EpiTYPER studies. To cope with the low amount of DNA, calibration assays will be required for each one.

The first large EWAS for adult obesity performed by Dick et al. [14] identified associations between three *HIF3A* CpGs (CpG1, CpG5, and CpG7 in our study) and body mass index. Although it was difficult to detect associations involving DNA methylation and gene expression in blood cells due to low expression levels in blood, an inverse correlation between methylation at cg22891080 (CpG5 in our study) and *HIF3A* expression in adipose tissues was found [14]. Pan et al. [13] postulated a role of *HIF3A* in acquired obesity, and demonstrated an association of *HIF3A* methylation in umbilical cords with birth size and adiposity at birth. Although the association of *HIF3A* methylation in cord blood cells with birth weight was not reported [20, 21], an association of such methylation using newborn blood spot screening cards with birth weight has been described [7]. We hypothesize that this is because *HIF3A* methylation is influenced by the proportion of nucleated blood cells, which uniquely exist in cord blood. Although Dijk et al. referred to an association between birth weight and *HIF3A* methylation using newborn blood spot screening cards, detailed information concerning effect size was not clearly reported. According to an EWAS report by Agha et al. [21], the effect size of BW/GA on methylation was in the range of 0.03–0.53% methylation change per 10% increment of BW/GA centile. Although our study found that the effect size of birth weight on methylation change was small (around 0.2% per unit increase in birth weight), this magnitude was within the range of that reported by EWASs for birth weight [21]. Thus, we reproduced the association of *HIF3A* methylation with birth weight by the secondary usage of post-test newborn blood spot screening cards.

Beyond the association with concurrent adiposity (birth weight or obesity), *HIF3A* methylation at birth potentially might have an additional value. Based on the DOHaD concept, antenatal environment may affect the developmental process of the fetus, which will alter his/her phenotype and disease susceptibility. DNA methylation has been suggested as the underlying mechanism by which early life exposures are transmitted to later life disease risk. It is plausible that *HIF3A* methylation is one of many epigenetic marks which respond to intrauterine environment. This idea is supported by several studies showing that gestational diabetes or pre pregnancy BMI indeed influenced cord blood *HIF3A* methylation [20, 22]. Therefore, methylation of *HIF3A* (combined with other markers) will be potentially useful for evaluation of intrauterine environment quality, and eventually for assessment of the future disease risk.

Several limitations of this study should be acknowledged. First, this study was limited to whole blood samples with a mixed cell composition. We did not examine the effect of cell type composition on *HIF3A* methylation. However, according to public databases, neonatal *HIF3A* methylation levels in lymphocytes, monocytes, and neutrophils, do not vary greatly. Therefore, it is likely that adjustment for cell type composition will not substantially change our conclusions. However, for the most other methylation sites, adjustment for cell type composition is required [23]. Future studies involving newborn blood spot screening cards will employ an epigenome-wide approach, which will enable the performance of estimation of cell type composition, with appropriate adjustments [23]. In this pilot study, only three attributes were identified. However, there are many potential confounders involved in BW/GA—DNA methylation association, such as maternal age, parity, mode of delivery, and other in utero environmental factors, which should be included for association studies. As advocated in [1], to implement life course health care and preemptive medicine, reorganization of our medical and social systems will be required. For the establishment of epidemiological studies, newborn screening biobanks, which store the residual dried blood samples, will be needed. Such model epidemiological research is currently possible in Denmark [12, 24]. The establishment of such biobanks, and accompanying exposome data collection, will be required for further epidemiological studies.

In conclusion, we demonstrated that post-test newborn blood spot screening cards retain DNA, in terms of sufficient quantity and quality, for establishing model epigenetic associations. Our data will contribute to the promotion of secondary use of post-test newborn blood spot screening cards to accelerate life course health research.

## Supplementary information

Supplementary Information
