# Supplementary material for: The utility of post-test newborn blood spot screening cards for epigenetic association analyses: association between HIF3A methylation and birth weight-for-gestational age
Source: J Hum Genet. 2019 May 29;64(8):795–801. doi: 10.1038/s10038-019-0621-5 (PMC6760750; doi:10.1038/s10038-019-0621-5)
Supplement: Supplementary file 1 — Supplementary Information [file 10038_2019_621_MOESM1_ESM.docx]

**Supplementary materials**

**The utility of post-test newborn blood spot screening cards for epigenetic association analyses: association between *HIF3A* methylation and birth weight**

Tay Zar Kyaw, Seiji Yamaguchi, Chihiro Imai, Marina Uematsu, and Noriko Sato

**Table S1.** Effect of DNA quantity employed for bisulfite conversion, followed by EpiTYPER assays

**Table S2.** Correlation matrix of *HIF3A* methylation

**Table S3.** Association between *HIF3A* methylation (each CpG unit) and birth weight-for-gestational age (BW/GA) centile in full-term pregnancies (n = 283)

**Table S4**. Association between *HIF3A* methylation and cis-genotypes (n = 283)

**Table S5**. Birth weight-for-gestational age (BW/GA) centile and cis-genotypes (n = 283)

**Figure S1.** Schematic diagram showing the *HIF3A* CpG units analyzed in this study

**Figure S2.** *HIF3A* methylation levels in newborn blood spot screening cards

**Figure S3.** Methylation differences among *HIF3A* diplotypes

**Table S1.** **Effect of DNA quantity employed for bisulfite conversion, followed by EpiTYPER assays**

**(1) The methylation measurement of the same adult blood sample with the different DNA amount**

| DNA amount employed (ng) | CpG1.2 | CpG5 | CpG6.7.8 | CpG11 | CpG13.14 |
| --- | --- | --- | --- | --- | --- |
| 50 | 45 (3.6) | 26 (3.3) | 52 (2.7) | 54 (4) | 42 (3.3) |
| 100 | 45 (2.7) | 28 (3.1) | 51 (2.6) | 51 (6.8) | 45 (2.9) |
| 150 | 47 (1.7) | 26 (3) | 52 (2) | 54 (3.2) | 43 (2.6) |
| 200 | 46 (4.9) | 28 (2) | 52 (3.4) | 54 (2.9) | 43 (2.3) |
| 500 | 49 (5.8) | 27 (2.8) | 52 (2.7) | 54 (3) | 42 (3.8) |
| 1000 | 47 (2.5) | 28 (1.9) | 51 (1.5) | 54 (2.8) | 45 (2.6) |

Data are mean (SD)

**(2) Correlation of the methylation measurement derived from different DNA amount**

| DNA amount to be bisulfite converted (ng) | 50 | 100 | 150 | 200 | 500 | 1000 |
| --- | --- | --- | --- | --- | --- | --- |
| 50 | 1.00 |  |  |  |  |  |
| 100 | 0.99 | 1.00 |  |  |  |  |
| 150 | 1.00 | 0.99 | 1.00 |  |  |  |
| 200 | 1.00 | 0.99 | 1.00 | 1.00 |  |  |
| 500 | 0.99 | 0.98 | 0.99 | 0.99 | 1.00 |  |
| 1000 | 0.99 | 0.99 | 1.00 | 1.00 | 0.99 | 1.00 |

**Table S2. Correlation matrix of *HIF3A* methylation**

|  | CpG5 | CpG6.7.8 | CpG11 | CpG13.14 |
| --- | --- | --- | --- | --- |
| CpG1.2 | 0.896 | 0.847 | 0.425 | 0.776 |
| CpG5 |  | 0.791 | 0.451 | 0.717 |
| CpG6.7.8 |  |  | 0.474 | 0.761 |
| CpG11 |  |  |  | 0.381 |

**Table S3. Association between *HIF3A* methylation and birth weight-for-gestational age (BW/GA) centile in full-term pregnancies (n = 283)**

|  | Unadjusted for cis-genotypes | | Adjusted for *cis*-genotypes | |
| --- | --- | --- | --- | --- |
|  | Est.  (95% CI) | *p* value | Est.  (95% CI) | *p* value |
| CpG1.2 | 0.23  (0.04-0.42) | 0.017 | 0.20  (0.03-0.36) | 0.023 |
| CpG5 | 0.24  (0.02-0.47) | 0.031 | 0.20  (0.00-0.40) | 0.045 |
| CpG6.7.8 | 0.17  (0.01-0.33) | 0.042 | 0.15  (-0.01 to 0.30) | 0.062 |
| CpG11 | 0.09  (-0.07 to 0.26) | 0.272 | 0.08  (-0.08 to 0.25) | 0.322 |
| CpG13.14 | 0.16  (0.00-0.32) | 0.052 | 0.14  (-0.01 to 0.29) | 0.076 |

Regression coefficients (Est.) and 95% confidential intervals (CIs) are reported as percentage change in methylation for one unit (10%) increase in birth weight-for-gestational age (BW/GA).

**Table S4. Association between *HIF3A* methylation and cis-genotypes (n = 283)**

|  | rs8102595 | | rs3826795 | |
| --- | --- | --- | --- | --- |
|  | Est.  (95% CI) | *p* value | Est.  (95% CI) | *p* value |
| CpG1.2 | 2.89  (2.14-3.64) | 4.47E-13 | 2.12  (1.46-2.78) | 9.95E-10 |
| CpG5 | 3.75  (2.89-4.62) | 1.03E-15 | 2.54  (1.76-3.32) | 5.71E-10 |
| CpG6.7.8 | 1.83  (1.16-2.51) | 1.98E-07 | 1.28  (0.69-1.87) | 2.83E-05 |
| CpG11 | 0.80  (0.09-1.51) | 2.67E-02 | 0.33  (-0.28 to 0.94) | 0.290 |
| CpG13.14 | 1.72  (1.04-2.39) | 9.74E-07 | 1.11  (0.53-1.7) | 2.40E-04 |

Regression coefficients (Est.) and 95% confidential intervals (CIs) are reported as percentage change in methylation per copy of the effect alleles G^a^ and G^b^.

**Table S5. Birth weight-for-gestational age (BW/GA) centile and cis-genotypes (n = 283)**

|  | rs8102595 | | rs3826795 | |
| --- | --- | --- | --- | --- |
|  | Est.  (95% CI) | *p* value | Est.  (95% CI) | *p* value |
| BW/GA centile | 2.22  (-2.89 to 7.33) | 0.394 | 0.80  (-3.58 to 5.18) | 0.719 |

Regression coefficients (Est.) and 95% confidential intervals (CIs) are reported as percentage change in BW/GA per copy of the effect alleles G^a^ and G^b^.

**Figure S1. Schematic diagram showing the *HIF3A* CpG units analyzed in this study**

*HIF3A* is a member of transcription factor family involved in response to hypoxia, but its function is still not well known. Human *HIF3A* gene is located on chromosome 19q13.2, spanning 43 kb and containing 19 exons. *HIF3A* has multiple transcript variants generated by alternative transcription initiation (among three initiation regions, two are CpG island (CGI) promoters and one is non-CGI promoter) and alternative splicing. The first EWAS by Dick et al^14^. and the subsequent EWAS by Pan et al^13^. reported that methylation at three CpG sites (cg27146050/CpG1, cg22891070/CpG5 and cg16672562/CpG7) adjacent to second transcription initiation region were associated with adult obesity and birth weight, respectively. The association of two nearby single nucleotide polymorphisms (rs8102595, rs3826795) with methylation was also found. In an EpiTYPER study, after bisulfite-treated DNA is amplified by PCR with using a primer tagged with a T7 promoter, the resulting PCR product is transcribed from the reverse strand. This RNA product is cleaved after each U (uridine), which results in a specific fragmentation of known mass. A fragment containing one or more CpG is called a CpG unit. In some cases, due to overlaps in mass among different fragments or out-of-detection range, the DNA methylation signal cannot be assigned to the specific CpG unit. In this *HIF3A* methylation assay, five CpG units (CpG1.2, CpG5, CpG6.7.8, CpG11, CpG13.14) were discernable. In the above figure, the positions of the SNPs in LD with rs8102595 (rs3810302, rs4803924, rs8112512, rs7252284, rs6509258, rs11669771, rs8100021, rs59642371) and with rs3826795 (rs60593560, rs28654980, rs11393349, rs11673299, rs56338777, rs2311053, rs8105156, rs2008030) are indicated by blue circles and purple triangles. The LD measure considered here is r-squared calculated using 1000GENOMES:phase_3:JPT.

**Figure S2. *HIF3A* methylation levels in newborn blood spot screening cards**

Methylation levels in each CpG unit are shown (n = 300). Data are presented as an aligned dot plot, and values of maximum, minimum, median and interquartile ranges are shown. Mean (standard deviation) of each CpG unit are CpG1.2, 73 (3.9); CpG5, 76 (4.6); CpG6.7.8, 79 (3.3); CpG11, 93 (3.4); CpG13.14, 66 (3.4).

**Figure S3. Methylation differences among *HIF3A* diplotypes**

Mean methylation levels of three CpG units (CpG1.2, CpG5 and CpG6.7.8) in each diplotype are shown. Methylation levels are increased as the number of either effect allele (rs8102595-G or rs3826795-G) increases. Data are presented as an aligned dot plot, and values of maximum, minimum, median and interquartile ranges are shown.
